# Development and Validation of a Next-Generation Time-Driven Activity-Based Costing Model for Quantifying Uncertainty: An Empirical Study of Fuzzy-Logic Time-Driven Activity-Based Costing Using Surgical System Data †

**DOI:** 10.3390/healthcare14203504

**Published:** 2026-10-18

**Authors:** Hirooki Yoshihara, Jaewook Kim

**Affiliations:** Graduate School of Humanities and Social Sciences, Hiroshima University, 1-1-89 Higashi-Senda-machi, Naka-ku, Hiroshima 730-0053, Japan; d244846@hiroshima-u.ac.jp

**Keywords:** fuzzy logic, time-driven activity-based costing (TDABC), surgical costs and uncertainty, operating room management, bundled payment (DPC/PDPS), value-based healthcare (VBHC), sensitivity analysis, validation

## Abstract

**Highlights:**

**What are the main findings?**

- Fuzzy rules built from four routinely recorded attributes of 4674 gastrointestinal surgeries gave cost intervals that covered 97.8% (observed range) and 76.5% (α = 0.5 cut) of realized costs in a two-year out-of-sample period.
- The fuzzy-logic time-driven activity-based costing (FL-TDABC) centroid exceeded realized cost by ¥36,080 per case (10.2%); decomposition and sensitivity analyses trace this premium to anchoring the fuzzy number on the observed maximum time, not to concealed cost or better accuracy.

**What are the implications of the main findings?**

- Cost uncertainty grows with surgical complexity in absolute terms (median α = 0.5 cost interval ¥51,000 in Rank 1 versus ¥282,000 in Rank 3), whereas the risk of a negative contribution margin concentrates in simple, low-fee procedures.
- Planning operating room time at the α = 0.5 upper bound instead of the mean would have cut overruns from 44% to 10% of cases at the cost of idle time; under fixed-fee reimbursement such as Japan’s Diagnosis Procedure Combination/Per Diem Payment System (DPC/PDPS), the α level is an explicit dial between overrun risk and capacity utilization.

**Abstract:**

Background/Objectives: Under fixed-fee reimbursement such as Japan’s Diagnosis Procedure Combination/Per Diem Payment System (DPC/PDPS), hospitals bear the cost of operations that run longer than planned, yet conventional time-driven activity-based costing (TDABC) reports a single cost per case. This study developed and validated a fuzzy-logic TDABC (FL-TDABC) model that expresses case-level time uncertainty as interpretable intervals. Methods: Data from 4674 gastrointestinal surgeries at a Japanese acute-care hospital (fiscal years 2020–2024) were analyzed. Four pre-operative attributes (surgical index rank, technical grade, number of anesthesiologists, and urgency) define 96 possible rules; for each rule, triangular fuzzy numbers for surgical, anesthesia, and room occupancy times were built from the observed minimum, mode, and maximum and defuzzified by the centroid method. Estimates were validated against realized costs in a temporal hold-out (rules from FY2020–2022 applied to FY2023–2024) and 5-fold cross-validation, and 21 alternative specifications were tested. Results: The FL-TDABC centroid exceeded realized cost by ¥36,080 per case (10.2%; Cohen’s d = 0.558). The premium arose entirely from the maximum-time vertex, vanished under percentile bounds, and did not improve point accuracy out of sample. The intervals were informative: the observed range of each rule covered the recorded room occupancy time of 97.5% of hold-out cases and the α = 0.5 cut 76%, interval width increased with surgical complexity, and planning room time at the α = 0.5 upper bound reduced overruns from 44% to 10% of cases. Conclusions: The value of FL-TDABC lies in interval information that can inform scheduling buffers, staffing, and risk assessment, not in a higher or more accurate point estimate.

## 1. Introduction

The global healthcare environment is undergoing rapid transformation, making timely adaptation a critical priority. Many Western countries have actively promoted value-based healthcare (VBHC), which emphasizes improving patient outcomes while enhancing cost efficiency [1]. To operationalize VBHC, time-driven activity-based costing (TDABC) has been widely advocated as a tool for improving cost transparency in healthcare processes [2,3,4,5]. TDABC enables more accurate and actionable cost measurement by linking resource consumption to process time.

For hospital managers and clinicians, however, the practical question is not only how much a surgical case costs on average, but how much its cost can vary and why. Operating rooms are among the most resource-intensive units of an acute-care hospital and a principal source of its revenue [6], and their performance depends on decisions that must be made under uncertainty: how many cases to schedule per room per day, how much buffer time to reserve, how to allocate anesthesiologists and nursing staff, and how to accommodate emergency cases without canceling elective surgery [7]. In Japan, surgical procedures are reimbursed at a fixed fee per procedure type under the national fee schedule, and most other inpatient costs are bundled under the Diagnosis Procedure Combination/Per Diem Payment System (DPC/PDPS) [8,9]. In neither case does reimbursement increase when an operation takes longer or consumes more resources than expected, so the financial consequences of time overruns, overtime staffing, and unplanned resource use are borne entirely by the hospital. Cost information that reports only mean values therefore leaves managers unable to see—let alone manage—the risk embedded in their surgical case mix.

Despite its conceptual advantages, the practical implementation of TDABC in clinical settings remains limited. The gap between clinical departments and administrative management leads to the underutilization of cost accounting information in decision-making. Moreover, although TDABC simplifies the traditional activity-based costing (ABC) framework, its standardization and applicability in healthcare contexts are constrained. TDABC relies on fixed time estimates, which inadequately capture the inherent variability and uncertainty of clinical workflows [10].

These limitations are especially pronounced in surgical settings, where cost and time variability are influenced by multiple interacting factors, including patient condition, procedural complexity, surgeon expertise, and case urgency. Consequently, conventional cost accounting approaches struggle to represent real-world surgical processes, limiting their usefulness for performance evaluation and resource allocation.

To address these challenges, recent studies have proposed integrating fuzzy logic into TDABC, leading to the development of fuzzy logic-based TDABC (FL-TDABC) models [11,12]. These models incorporate fuzzy representations of time and help capture variability and uncertainty in clinical processes. However, research has largely remained at the conceptual or simulation level, with limited empirical validation using large-scale clinical data.

Fuzzy logic is particularly well suited to this problem in healthcare delivery for four reasons. First, surgical process times are right-skewed and bounded [13]; a triangular fuzzy number, defined by a minimum, a most likely, and a maximum value [14], represents exactly the way surgeons and operating room coordinators already describe a case (“usually about two hours, occasionally three”), whereas a single mean does not [15]. Second, many clinically meaningful case categories contain few observations [16], and a fuzzy number can be specified from sparse data or expert judgment, whereas fitting probability distributions requires sample sizes that rare case types never reach [12,17]. Third, the fuzzy rules are expressed in the variables that surgical teams use when planning—surgical index rank, technical grade, number of anesthesiologists, and urgency—so the resulting cost information is interpretable to clinicians as well as to administrators. Fourth, the outputs are intervals that translate directly into scheduling buffers and staffing margins, and all inputs are routinely captured by surgical management systems, so no additional time measurement—a well-documented barrier to TDABC implementation in hospitals [18,19]—is required.

This study develops and empirically applies an FL-TDABC model using real-world surgical system data.

This study makes three contributions. First, using 4674 surgical cases, it shows that fuzzy rules built from four routinely recorded pre-operative attributes yield case-level cost intervals with a defined empirical meaning: in a two-year out-of-sample period, the observed range of each rule covered the recorded room occupancy time of 97.5% of cases and the α = 0.5 cut 76%. Second, it explains the widely reported finding that fuzzy costing yields “higher” or “more realistic” costs: the FL-TDABC centroid exceeded realized cost by ¥36,080 per case (10.2%), but this premium comes from the maximum-time vertex of the triangular fuzzy number, disappears under percentile bounds, and does not improve accuracy—it is a property of the specification, not hidden cost. Third, it shows how the intervals can be used under fixed-fee reimbursement: to set operating room block times with an explicit overrun–idle trade-off, to identify the case groups in which margin risk concentrates, and to relate cost variability to its drivers with explainable machine learning. FL-TDABC thus complements conventional TDABC by adding a range with known coverage to a point estimate.

This paper proceeds as follows. Section 2 outlines our research objectives and questions, and Section 3 reviews the relevant literature. Subsequently, Section 4 details the research methodology, and Section 5 presents the analytical results. Finally, Section 6 discusses the findings and their implications for healthcare delivery, and Section 7 presents the conclusions. An earlier version of this work was presented at the APMAA 2025 Annual Conference [20]; this article extends it with the validation, sensitivity, and stratified analyses reported below.

## 2. Research Objectives

Delivering high-quality care with limited resources is a pressing challenge, especially in Japan, where population aging and rising healthcare expenditure coincide. In fiscal year 2022, national medical expenditure reached ¥46.7 trillion, equivalent to 8.24% of gross domestic product [21]. In response to sustained cost pressure, Japan introduced the Diagnosis Procedure Combination/Per Diem Payment System (DPC/PDPS) in 2003 [8,9], under which effective cost management has become indispensable in a bundled payment environment.

In this context, VBHC, which balances clinical outcomes and costs [1], has gained attention, and case-level cost information has become critical for hospital decision-making. In Japan, successive reimbursement revisions and the spread of the DPC have intensified the need for management accounting tools that relate outcomes to costs. The need is most evident in surgical departments, which are central to hospital revenue yet highly variable: complexity, patient condition, staffing, anesthesia method, and urgency jointly affect time, cost, and outcomes, and even under standardized protocols, surgical, anesthesia, and room occupancy times vary in ways that averages cannot represent.

The conventional TDABC model, which relies on fixed estimates of activity time and unit cost, is limited in its ability to capture such variability and nonlinear influences. The use of average time values may misestimate individual cases, particularly complex or atypical ones.

To address these limitations, this study develops and empirically validates a cost accounting model that explicitly incorporates clinical variability and uncertainty. Specifically, it proposes an FL-TDABC model that relaxes the deterministic assumptions of conventional TDABC by modeling activity time using triangular fuzzy numbers (TFNs) and applying the centroid method for defuzzification.

The model is built in three steps: (1) fuzzy rules are defined by combinations of four pre-operative variables—surgical index rank, technical grade, number of anesthesiologists, and urgency status (3 × 4 × 4 × 2 = 96 possible rules, of which 39 occurred); (2) for each rule, TFNs for surgical, anesthesia, and room occupancy times are defined by the minimum, most frequent, and maximum observed values; (3) the centroid method converts each fuzzy time into a representative value for the cost calculation, and α-cuts provide interval estimates. The study examines both the point estimate and the interval against realized costs.

Based on these perspectives, this study formulates three research questions (RQs) to evaluate the effectiveness and generalizability of the model.

RQ1 (Methodological Validity):

How can the uncertainty in the surgical process be quantified and integrated into cost accounting? How does the use of TFNs and the centroid method influence the representation of time-related variability and uncertainty compared to the conventional fixed-value TDABC model?

RQ2 (Multidimensional Influence Structure):

How do the four factors—surgical index rank, technical grade, number of anesthesiologists, and urgency status—affect cost structure? To what extent can the FL-TDABC model quantitatively capture these complex interrelationships?

RQ3 (Decision-Support Value):

What value does the FL-TDABC model add to decision-making—operating room scheduling, contribution margin analysis, and resource allocation—compared with the conventional TDABC model, and how do its point estimates and intervals perform against realized costs out of sample?

Taken together, RQ1 asks whether uncertainty can be represented in a form that hospitals can use, RQ2 asks which clinically meaningful factors drive that uncertainty, and RQ3 asks whether the resulting information can inform the decisions hospitals make about operating room resources; the three questions thus move from measurement to healthcare delivery performance.

## 3. Literature Review

The TDABC model has attracted attention as a managerial accounting method that simplifies implementation compared to the traditional ABC model and enables detailed cost visualization at the activity level. In the healthcare sector, the TDABC model has been increasingly introduced as a tool for achieving structural cost comprehension and operational efficiency in highly complex and individualized processes. In surgical care, numerous studies have sought to optimize resource use and reduce costs by measuring process time and visualizing surgical workflows. These studies suggest that the theoretical and practical development of the TDABC model in healthcare has progressed in three stages: introduction, application, and advancement.

During the initial phase of introduction and foundational implementation (2015–2018), the TDABC frameworks were adapted to clinical settings, with their effectiveness in visualizing the structure of surgical and clinical workflows being preliminarily validated. According to Balakrishnan et al. [22], the TDABC model could reduce staff costs by 23.2% when a single surgeon operates two rooms simultaneously, indicating the TDABC model’s utility in optimizing resource allocation. Yu et al. [23,24] visualized cost contribution rates by department, such as the operating room, wards, and emergency units, for pediatric appendicitis cases and reported a 51% reduction in length of stay and an 11% reduction in total costs through process improvements. In the subsequent phase of application and managerial integration (2018–2021), the TDABC model’s use expanded to support strategic decision-making, particularly in managing complex care processes. Andreasen et al. [25] compared the joint replacement surgery processes at two hospitals and argued that fast-track surgery was more cost-efficient than conventional methods. According to Husted et al. [26], outpatient total hip and knee arthroplasty procedures can be performed at approximately two-thirds of the cost of inpatient operations.

In the last phase of advancement and digital integration (2021–present), the TDABC model has evolved into a next-generation tool that enables real-time, highly accurate cost management through integration with information and communications and artificial intelligence (AI) technologies. Kellett et al. [18] argued that healthcare workers could self-record activity time with accuracy comparable to that of traditional observation methods using a smartphone application called “dTool.” Etges et al. [19] leveraged electronic health records (EHR), timestamping, and AI-based automation to improve the accuracy and speed of data collection and analysis within the TDABC model; they explored its potential to optimize supply costs and staffing allocations.

Nevertheless, the TDABC model has theoretical limitations. It struggles to address “uncertainty” and “nonlinearity” in surgical procedures, which are inherently variable and difficult to predict. Fixed estimates of time and resource inputs often fail to indicate the complexity of care delivery, generating a growing interest in integrating fuzzy logic with the TDABC model to create an FL-TDABC model. Fuzzy set theory, originally proposed by Zadeh [14], provides a mathematical framework for handling ambiguity and subjectivity, enabling rational estimation and decision-making, even under uncertain conditions.

A fuzzy number is reduced to a single value by a defuzzification method—centroid, bisector of area, mean of maximum, or a weighted average of the vertices—and these methods give systematically different values for asymmetric membership functions [27]. Three-point estimation from minimum, most likely, and maximum values has a long history in project planning [28] and is the form most FL-TDABC applications adopt because it can be specified from sparse data. How much cost estimates depend on these choices has rarely been examined empirically.

Esmalifalak et al. [17] compared the ABC, Fuzzy ABC, and Monte Carlo-based ABC models and discovered that the Fuzzy ABC model delivered the most accurate cost estimates under high uncertainty. Ostadi et al. [12] proposed an FL-TDABC model for hospital services that incorporated fuzzy logic into the TDABC model using TFNs to represent uncertainties in time, capacity, and cost mathematically. Their findings indicated that the FL-TDABC model achieved more realistic and flexible cost estimations than the conventional TDABC model, particularly in high-variability departments, such as blood banks and microbiology laboratories.

This study follows this line of development by constructing and validating an uncertainty-aware cost-estimation model.

Concomitantly, recent research has begun to examine alternative cost accounting methods, such as the performance-focused ABC (PFABC) model, by comparing them to the TDABC and FL-TDABC models. The PFABC model emphasizes performance metrics, such as efficiency and outcomes, in addition to activity units and time input—the focus of the traditional ABC and TDABC models. It visualizes resource allocation and evaluates the results achieved with those resources, balancing cost appropriateness with healthcare quality.

However, the PFABC model presents practical and theoretical challenges—difficulties defining objective performance indicators, the subjective nature of the evaluation criteria, and the substantial data required for quantification. The PFABC model may face institutional barriers to implementation, particularly in countries such as Japan, where reimbursement systems are based on procedures rather than outcomes.

Accordingly, a theoretical comparison of the TDABC, FL-TDABC, and PFABC models can be summarized as follows. The TDABC model clarifies the causal relationships between time and cost and is effective for process standardization and efficiency evaluation. Nevertheless, its ability to incorporate uncertainty factors and qualitative outcomes remains limited. Conversely, the FL-TDABC model represents variability and ambiguity as fuzzy sets, which is particularly useful in settings with high variability and diverse procedures, where interval estimation using TFNs (minimum, mode, and maximum) provides richer cost information than a single mean-based estimate.

Contrarily, the PFABC model is innovative in its integrative assessment of costs and outcomes. Nonetheless, owing to its dependence on data quality and institutional systems, it remains largely in the developmental phase. Therefore, currently, for practical implementation in healthcare organizations, a realistic approach is to use the TDABC model as a foundation and incorporate the FL-TDABC model to address variability and uncertainty.

## 4. Materials and Methods

### 4.1. Setting and Data Sources

The study was conducted at a core acute-care hospital (hereafter referred to as Hospital A), which was anonymized in accordance with ethical considerations.

Hospital A is a regional hub medical institution with approximately 400 acute-care beds, 26 clinical departments, and 8 operating rooms. Furthermore, it is a designated cancer treatment center and maintains a 24/7 emergency care system.

The target dataset comprised surgical cases from the general gastrointestinal surgery department performed over five fiscal years (April 2020–March 2025; hereafter FY2020–FY2024). This department was selected for three reasons: (1) the number of cases was stable throughout the year, ensuring sufficient statistical robustness; (2) the surgical procedures were relatively standardized, enabling meaningful comparisons; and (3) the surgical system maintained detailed and structured records, including time logs and staffing information. In April 2023 the department was renamed from General Surgery to Gastrointestinal Surgery, with no change in staff, services, or facilities.

Initially, data were collected using Hospital A’s surgical management system and EHR files. The cost-related data, extracted from the surgical management system, included detailed, minute-level logs for each case, such as “surgical duration,” “anesthesia time,” and “room occupancy time,” along with staff assignment information (e.g., surgeon, anesthesiologist, nurses), and medical device/material usage. Revenue information was obtained from the EHR files, which reflect insurance reimbursement points and allow income estimates based on surgical procedure fees. Importantly, all model inputs are routinely recorded in the course of care delivery; no additional time-and-motion observation was required, which is essential for the practical adoption of the model in hospitals where measurement burden has limited TDABC implementation [18,19].

Of the 4738 records extracted, 64 (1.4%) lacked a Gaihoren technical grade and surgical index—including two endoscopic procedures listed in a separate schedule—and were excluded, leaving 4674 cases. Surgical classification followed the Gaihoren Shian (proposal for surgical fee schedules) of the Federation of Surgical Societies’ Health Insurance Committees of Japan (Gaihoren) [29,30,31]: each case received a “surgical index”, a composite score reflecting duration, personnel, and complexity, and a “technical grade” (B–E) representing specialization and risk.

The dataset was integrated by case ID and screened for (1) room occupancy time shorter than anesthesia or surgical time, (2) implausible process times, and (3) missing values; no case failed, and no extreme values were trimmed, so all fuzzy-number bounds reported below are observed values. Analyses were performed in Python 3.13.16 (Anaconda Distribution) using pandas 2.2.3, NumPy 2.1.3, SciPy 1.16.3, Matplotlib 3.10.0, scikit-learn 1.6.1, XGBoost 3.4.1, and Shapley Additive exPlanation (SHAP) 0.52.0.

An FL-TDABC model was then constructed to estimate surgical costs and incorporate the variability and uncertainty inherent in surgical workflows. This model uses TFNs to model uncertainty in process time, so that time-related uncertainty is represented explicitly rather than by the deterministic “time × unit cost” structure of the conventional TDABC framework.

The study protocol was approved by the Research Ethics Committee of the Graduate School of Humanities and Social Sciences, Hiroshima University (Research Ethics Clearance No. HR-LPES-002040; 23 August 2024). Individual informed consent was waived because only retrospective, anonymized records were used, and the hospital gave written consent for the research use of the data.

### 4.2. Rule Variables

Four pre-operative variables define the rule antecedents (Table 1): surgical index rank, technical grade, number of anesthesiologists, and urgency. They were selected from the variables in the surgical management system by four criteria: known before the operation, so that a rule can be applied prospectively; recorded for every case without manual collection; a clinically meaningful ordinal or categorical scale; and related to both resource use and revenue under the Japanese fee schedule. The surgical index and technical grade come from the Gaihoren Shian [29,30,31], the survey-based classification that underlies fee-schedule proposals: the index is a composite of typical duration, personnel, and complexity, and the grade (B–E) expresses the skill required. The number of anesthesiologists reflects the anesthesia staffing model and determines anesthesia labor cost; urgency (scheduled versus emergency or ad hoc) captures the preparation, off-hours, and coordination burden of unplanned cases. Process times, the numbers of surgeons and nurses, and material use are outcomes of the operation rather than planning attributes and enter the model as fuzzy consequents or cost inputs; surgeon identity was excluded because surgeon-specific rules would be too sparse and would not transfer across institutions; and patient-level clinical characteristics were not available in the surgical management system extract. The surgical index was divided into three ranks by its distribution (<14.10, 14.10–31.73, ≥31.73), technical grade took the official values B–E, the anesthesiologist count ranged from 0 to 3, and urgency was scheduled versus other (emergency and ad hoc), giving 3 × 4 × 4 × 2 = 96 possible rules, of which 39 occurred (Section 5.1).

### 4.3. Triangular Fuzzy Numbers and Defuzzification

For each process time (surgical, anesthesia, and room occupancy) and each rule, a TFN (*a*, *m*, *b*) was defined from the data: *a* is the minimum, *m* the mode (the most frequent value in minutes; among ties, the first in data order), and *b* the maximum observed within the rule. No expert judgment, trimming, or smoothing was applied, so each fuzzy number reproduces the full observed range of its rule and can be updated automatically from the surgical management system. The TFN was chosen because it requires the fewest parameters and can be specified for sparse rules; its three points match how surgical teams describe a case (“at least, usually, at most”) [28]; it is the form used in previous FL-TDABC studies [11,12]; and its centroid, (*a* + *m* + *b*)/3, equals the mean of a triangular distribution with the same vertices, which gives the defuzzified value a transparent interpretation. The mode was used as the representative value because it reflects the most common operational reality in right-skewed time distributions; because the mode of continuous data can be unstable, the median, 10% trimmed mean, and mean were examined as alternatives (Section 4.5). Fuzzy times were defuzzified by the centroid method, and α-cuts, [*a* + α(*m* − *a*), *b* − α(*b* − *m*)], provided interval estimates at α = 0.5 and 0.9. Rules with fewer than 10 cases (14 of the 39 observed rules; 59 cases, 1.3%) were retained as observed in the base analysis; hierarchical pooling, in which such a rule borrows its TFN from the parent group obtained by collapsing, in order, urgency, number of anesthesiologists, and rank (and, if necessary, pooling all cases), was evaluated in the sensitivity and validation analyses. The TFNs of all 39 observed rules are listed in Appendix A (Table A1).

### 4.4. Cost Calculation Framework

Case-level costs comprise three labor pools—surgeons, anesthesiologists, and operating room nurses—plus materials and drugs. Following TDABC [2,3], the authors derived a capacity cost rate per minute for each pool and fiscal year from the hospital’s personnel expense records, as annual personnel expense of the pool divided by practical capacity, defined as full-time-equivalent staff × contracted working days (243–244) × 7.5 h × 60 min (Table 2). The rates were ¥133–141 per surgeon-minute, ¥97–120 per anesthesiologist-minute, and ¥51–52 per nurse-minute, rising with salary revisions. Operating room overhead (equipment, building, and administration) was not allocated, so the figures are direct costs. Material and drug costs were the actual consumption recorded for each case, and revenue was the sum of the procedure, anesthesia, and material fees claimed (fee-schedule points × ¥10) in the reimbursement records described in Section 4.1.

For case *i* in fiscal year *y* with recorded surgical, anesthesia, and room occupancy times *t_i_*^S^, *t_i_*^A^, and *t_i_*^R^, and *n_i_*^S^ surgeons, *n_i_*^A^ anesthesiologists, and *n_i_*^N^ nurses (scrub and circulating), the realized TDABC cost is

*C_i_*^TDABC^ = *M_i_* + *t_i_*^S^·*r*_S,*y*_·*n_i_*^S^ + *t_i_*^A^·*r*_A,*y*_·*n_i_*^A^ + *t_i_*^R^·*r*_N,*y*_·*n_i_*^N^, (1)

where *M_i_* is the material and drug cost and *r*_S,*y*_, *r*_A,*y*_, and *r*_N,*y*_ are the capacity cost rates in year *y*. FL-TDABC replaces each recorded time by the defuzzified time *τ* of the rule *k*(*i*) to which the case belongs,

*C_i_*^FL^ = *M_i_* + *τ_k_*_(*i*)_^S^·*r*_S,*y*_·*n_i_*^S^ + *τ_k_*_(*i*)_^A^·*r*_A,*y*_·*n_i_*^A^ + *τ_k_*_(*i*)_^R^·*r*_N,*y*_·*n_i_*^N^, (2)

where *τ* = (*a* + *m* + *b*)/3 is the centroid of the rule’s fuzzy time, and the α-cut cost interval follows by substituting the bounds of the α-cut of each fuzzy time for *τ*. For validation, a mean-based (standard-time) TDABC estimate replaces *τ* with the mean recorded time of the rule—the conventional ex ante TDABC estimate when case-level times are not yet known. Contribution margin is revenue minus cost. Under Equations (1) and (2), the per-case difference between the models depends only on the process times,

Δ*C_i_* = Σ*_p_ r_p_*_,*y*_·*n_i_^p^*·(*τ_k_*_(*i*)_*^p^* − *t_i_^p^*),     *p* ∈ {S, A, R}, (3)

so the mean difference decomposes into the contributions of the minimum, mode, and maximum vertices, *r*·*n*·(*a* − *t*)/3 + *r*·*n*·(*m* − *t*)/3 + *r*·*n*·(*b* − *t*)/3. For example, for the most common rule for complex procedures (Rank 3, grade D, one anesthesiologist, scheduled; *n* = 796), the room occupancy TFN was (148, 337, 1010) min, with centroid 498 min against a recorded mean of 373 min and an α = 0.5 cut of [243, 674] min.

### 4.5. Comparative Analysis, Sensitivity Analysis, and Validation

The models were compared at several levels. First, point estimates were compared case by case with paired t-tests and Cohen’s d for paired differences [32], because significance is nearly guaranteed with 4674 pairs; results were stratified by technical grade and rank, and the mean difference was decomposed by TFN vertex. Second, sensitivity to the fuzzy specification was assessed by re-estimating all costs under 21 alternatives: the representative value (mode, median, 10% trimmed mean, mean); the support (observed minimum–maximum; 2.5th–97.5th, 5th–95th, and 10th–90th percentiles); the membership function and defuzzification method (triangular centroid, trapezoid with a plateau between the 25th and 75th percentiles, weighted average (*a* + 2*m* + *b*)/4, bisector of area, and mean of maximum) [27]; ±10–20% perturbations of the vertices; and hierarchical pooling of rules with fewer than 10 or 30 cases. For each, the mean cost, the mean and relative difference from realized cost, the share of cases with a higher estimate, Cohen’s d, and Spearman’s ρ of case-level costs and differences against the base specification are reported.

Third, the model was validated against realized resource use. In a temporal hold-out, rules were estimated from FY2020–FY2022 (*n* = 2807) and applied to FY2023–FY2024 (*n* = 1867); rules unobserved in the training period (two rules, five cases) received pooled parameters. The point estimates of FL-TDABC, the mean-based TDABC estimate, and a median-based estimate were compared with realized cost by bias (mean error), mean absolute error (MAE), root mean squared error (RMSE), and median absolute percentage error. The intervals were evaluated by empirical coverage—the proportion of hold-out cases whose recorded times and realized cost fell within the support and within the α = 0.5 and α = 0.9 cuts—and by their use for scheduling: the room time that would have been planned under each rule (rule mean, FL-TDABC centroid, or the α-cut upper bound for α from 0 to 1) was compared with the recorded time to obtain the overrun rate, mean overrun, and mean idle time. Rolling one-year-ahead validation and 5-fold cross-validation served as robustness checks. Fourth, temporal variation was examined by comparing case mix, process times, unit rates, and cost differences across fiscal years (Kruskal–Wallis tests) and by re-estimating the rules within each year. Fifth, the relationship between complexity and uncertainty was quantified by the width of the α = 0.5 time and cost intervals by rank (Spearman’s ρ, Kendall’s τ, and Kruskal–Wallis tests), the coefficient of variation of recorded times, and the proportion of cases for which the lower margin bound (revenue minus the upper cost bound) was negative. Finally, the drivers of margin variation were identified with SHAP [33] applied to an XGBoost regression of contribution margin on process times, staffing, unit rates, and rule variables. The code for the sensitivity and validation analyses was prepared with the assistance of Claude (Claude Fable 5.1; Anthropic, San Francisco, CA, USA; 2026) and was verified by the authors by reproducing the previously reported results from the source data.

## 5. Results

### 5.1. Case Distribution and Rule Coverage

The basic descriptive statistics of 4674 surgical cases revealed a positively skewed distribution of surgical, anesthesia, and room occupancy times. The average surgical time was 156.0 min (SD: 116.8, min: 4.0, max: 907.0), anesthesia time averaged 205.3 min (SD: 132.2, min: 5.0, max: 995.0), and room occupancy time averaged 221.1 min (SD: 133.7, min: 15.0, max: 1010.0). These distributional characteristics motivated the use of the mode as the representative value in the centroid method of the FL-TDABC model: unlike the mean or median, the mode reflects the most frequently observed value in practice. The sensitivity of the results to this choice is examined in Section 5.3.

Analysis of the relationship between the surgical index rank and technical grade revealed that grades B and C were predominant (16.2% and 63.6%, respectively) in rank 1 (comprising 34.3% of all cases), whereas grade D was overwhelmingly dominant (99.7% and 98.5%, respectively) in ranks 2 and 3 (40.3% and 25.4%, respectively). Grade E was observed in only 18 patients (0.4%). Thus, the three-tiered surgical index classification reflects procedural complexity through intentionally uneven segmentation. Table 3 shows the distribution of the 4674 cases across the rule variables and fiscal years. Of the 96 possible rules, 39 occurred (Table A1): 25 rules with at least 10 cases held 98.7% of cases, 10 rules had 2–9 cases, and 4 rules had a single case (for which the three vertices coincide and the fuzzy estimate equals the recorded time). The two largest rules—scheduled grade D procedures with one anesthesiologist in Rank 2 (*n* = 925) and Rank 3 (*n* = 796)—held 37% of cases. Median room occupancy time rose steeply across ranks (108, 193, and 356 min), whereas its coefficient of variation fell from 0.52 in Rank 1 to 0.35 and 0.34 in Ranks 2 and 3.

### 5.2. Comparison of Point Estimates

The mean FL-TDABC cost was ¥390,943, ¥36,080 (10.2%) above the mean realized TDABC cost of ¥354,863 (Table 4); the difference was significant (paired t = 38.15, *p* < 0.001), varied widely across cases (SD ¥64,663), had a medium effect size (Cohen’s d = 0.558), and was positive in 80.7% of cases, whereas the mean-based TDABC estimate reproduced realized cost on average (−¥1859). Decomposition by TFN vertex (Equation (3)) explains the premium: the minimum and mode vertices contributed −¥27,674 and −¥9427 per case, the maximum vertex +¥73,181. In right-skewed time distributions the observed maximum lies far above the mode—for room occupancy, the case-weighted rule minimum, mode, and maximum averaged 95, 188, and 563 min against a recorded mean of 221 min—so the centroid (282 min) exceeds the recorded mean, and the premium is the price of giving the extreme upper bound one-third of the weight. The difference is thus an expected-value difference under an implied triangular distribution with the observed support, not evidence of greater accuracy (Section 5.3 and Section 5.4). Aggregated over the 4674 cases, it amounts to ¥168.6 million over five fiscal years under the base specification, and the effect sizes in Table 4 and Table 5 show that it varies with case characteristics.

Stratified analysis by technical grade and surgical index rank (Table 5) revealed that the upward adjustment was largest in D-Rank 3 cases (+¥61,912 per case; *n* = 1168) and D-Rank 2 cases (+¥39,360; *n* = 1880), and that the contribution margin was lower under FL-TDABC than under TDABC in every major case group. The only exception was the small C-Rank 2 group (*n* = 5; −¥46,272), which illustrates that the adjustment is bidirectional and depends on the shape of each group’s time distribution. Scatterplot analysis confirmed that in 80.7% of the cases, the FL-TDABC model estimated higher costs than the TDABC model.

**Figure 1:**
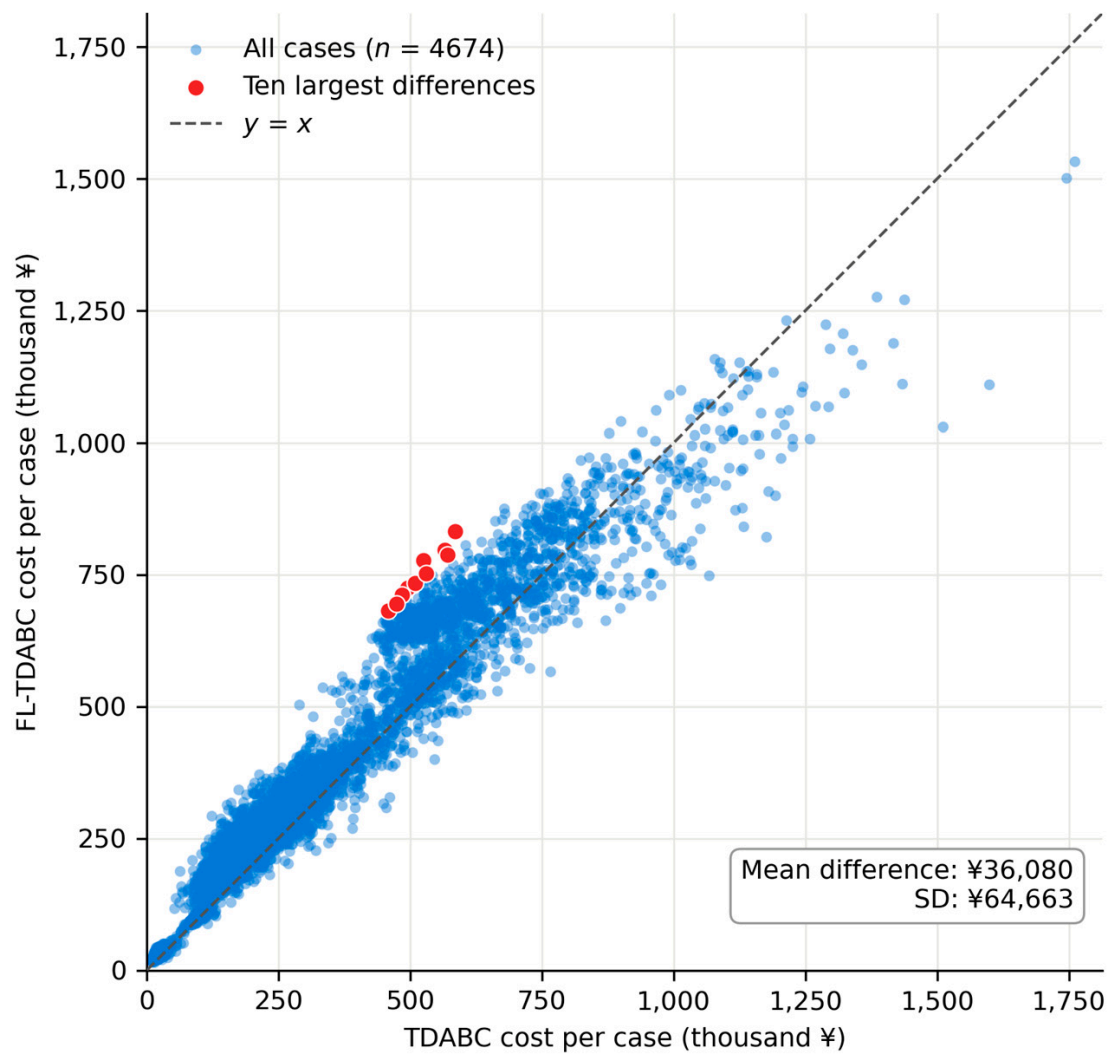
Comparison between TDABC and FL-TDABC estimated costs (scatter plot). Blue, all cases; red, the ten largest differences; dashed line, y = x.

Figure 1 plots the FL-TDABC model against the TDABC values to compare the cost estimates visually. In total, 80.7% of the cases fell above the diagonal (y = x), demonstrating a consistent tendency for the FL-TDABC model to yield higher cost estimates. In the ¥500,000–¥750,000 range, divergence was particularly prominent, owing to the wide observed range of moderately complex surgeries, which enters the fuzzy estimate through the upper vertex.

The red-highlighted cases are those whose recorded times lay far below the upper bound of their rule; their fuzzy estimate is high because the rule contains long cases, not because TDABC underestimated their own resource use.

For procedures prone to prolonged times, the fuzzy estimate thus carries an allowance for the upper tail that a point estimate does not; whether this allowance is useful depends on the decision it informs (Section 5.4).

### 5.3. Sensitivity to the Fuzzy Specification

**Figure 2:**
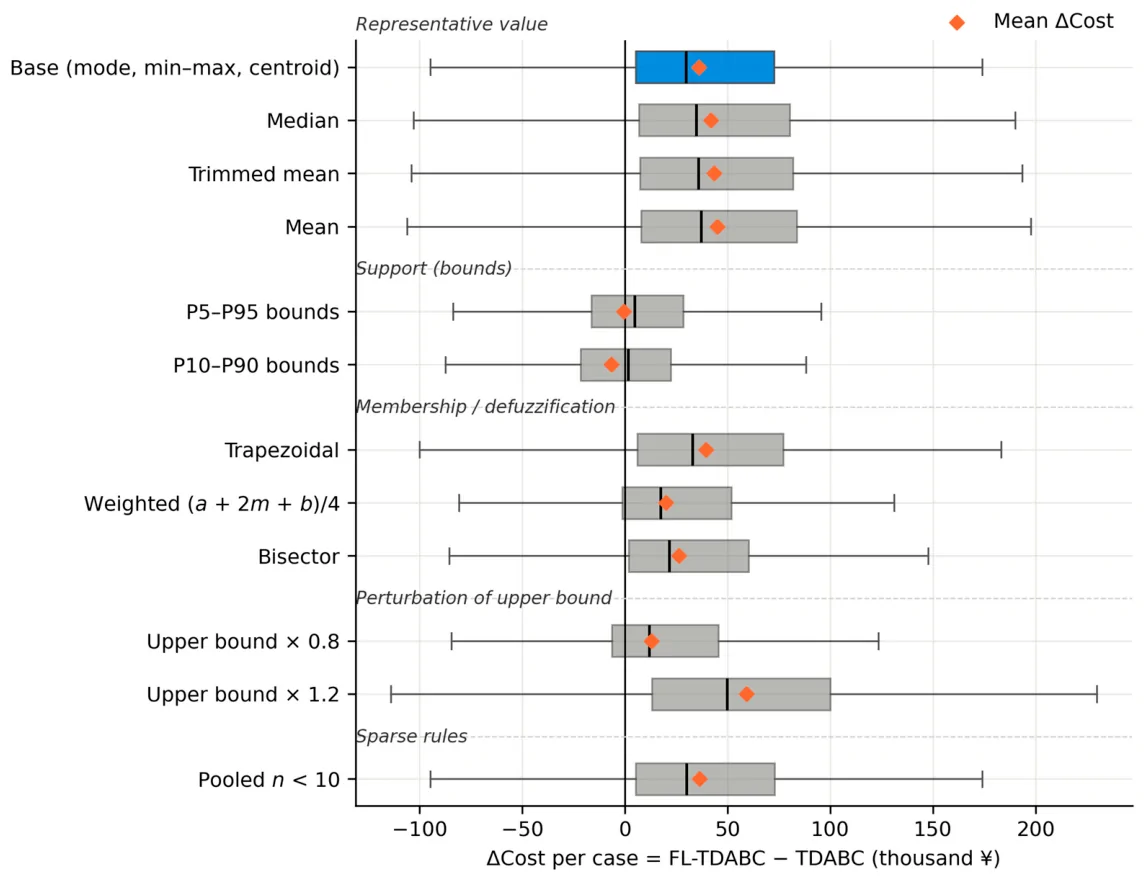
Sensitivity of the case-level cost difference (ΔCost = FL-TDABC − TDABC) to the fuzzy specification. Boxes show the interquartile range and whiskers 1.5 × IQR across the 4674 cases (outliers omitted); diamonds show the mean. The base specification (mode, observed minimum–maximum, centroid) is in blue; P5–P95 and P10–P90 denote supports defined by the 5th–95th and 10th–90th percentiles of each rule; “Pooled *n* < 10” assigns rules with fewer than 10 cases the parameters of their parent group.

Table 6 and Figure 2 report the 21 alternative specifications. The representative value mattered little: with the median, 10% trimmed mean, or mean in place of the mode, the mean difference from realized cost was 11.8%, 12.2%, and 12.6%, and case-level rankings were preserved (ρ ≥ 0.99). Pooling sparse rules changed the mean difference by less than 0.1 percentage points (10.2% at both thresholds), so the 59 cases in sparse rules do not drive the results. The support, by contrast, was decisive: replacing the observed extremes by the 2.5th–97.5th, 5th–95th, and 10th–90th percentiles reduced the mean difference to 1.5%, −0.2%, and −1.9%, and scaling the upper vertex alone by 0.8 or 1.2 moved it to 3.6% and 16.7%. The defuzzification method also mattered: the weighted average (*a* + 2*m* + *b*)/4 gave 5.6%, the bisector of area 7.4%, the trapezoidal membership 11.1%, and the mean of maximum (the mode itself) −8.0%. Case-level cost rankings were robust under every specification (ρ ≥ 0.987), whereas the ranking of case-level differences was moderately sensitive to the support (ρ = 0.80–0.88). The direction and size of the premium thus depend on how the upper bound is defined and weighted; the ordering of cases by cost does not.

### 5.4. Validation Against Realized Costs

Table 7 reports the temporal hold-out, in which rules estimated from FY2020–FY2022 were applied to the 1867 cases of FY2023–FY2024. As a point estimator, FL-TDABC was biased upward (+¥23,024 per case) and less accurate than the mean-based TDABC estimate (MAE ¥52,938 versus ¥42,515; median absolute percentage error 13.4% versus 9.2%); the median-based estimate was biased downward (−¥16,707) with an MAE similar to the mean-based estimate. The 5-fold cross-validation gave the same ordering (MAE ¥53,546 versus ¥38,426), as did the rolling one-year-ahead validation in each of FY2021–FY2024 (FL-TDABC MAE ¥45,463–61,604 versus ¥34,219–43,904 for the mean-based estimate), in which the α = 0.5 cut covered 76–79% of recorded room occupancy times each year and the support 96–98%. The FL-TDABC point estimate therefore does not improve accuracy.

**Figure 3:**
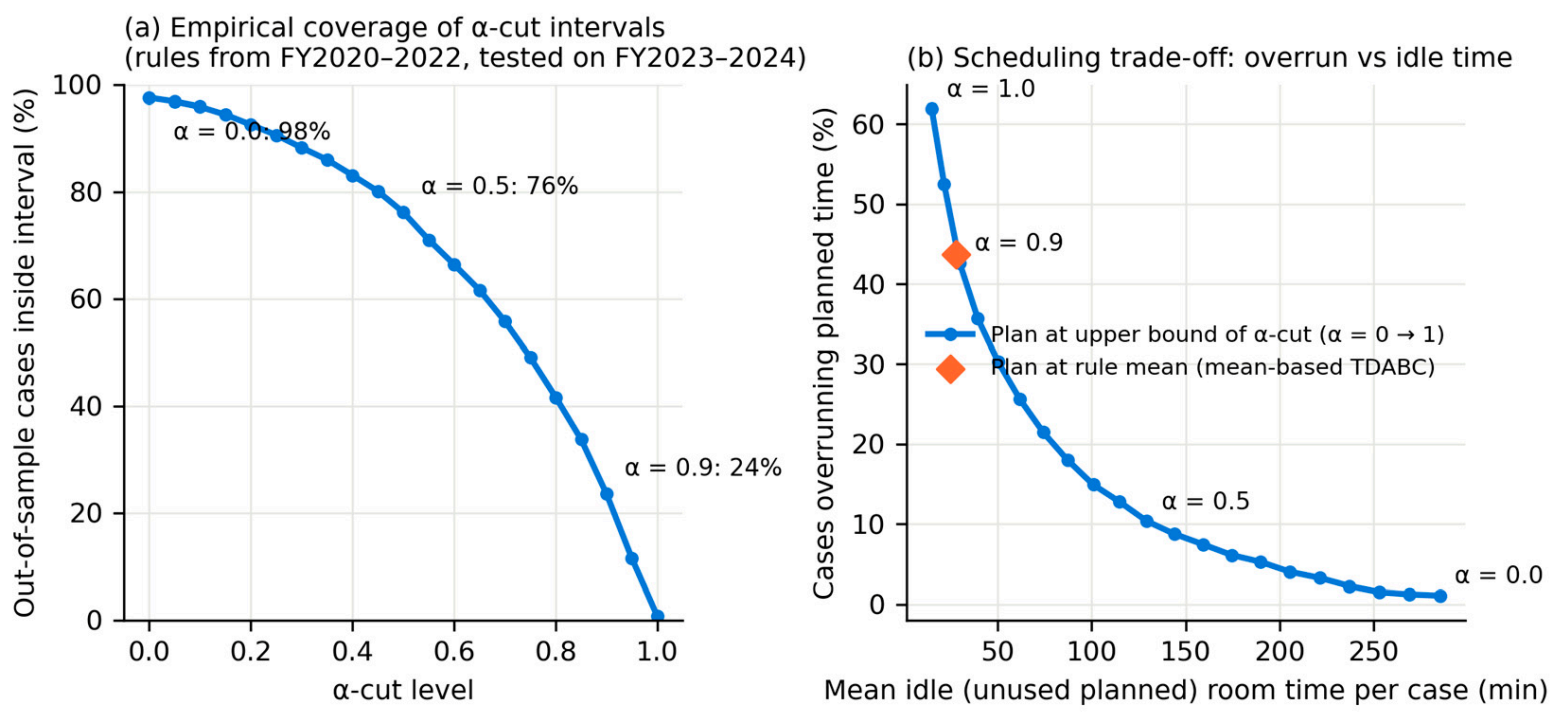
Temporal hold-out validation (rules estimated from FY2020–FY2022 and applied to the 1867 cases of FY2023–FY2024). (**a**) Proportion of hold-out cases whose recorded room occupancy time fell within the α-cut of their rule, by α (α = 0, observed range; α = 1, mode). (**b**) Overrun rate versus mean idle time per case when room occupancy time is planned at the upper bound of the α-cut (blue; α from 0 at the lower right to 1 at the upper left) or at the rule mean, i.e., the mean-based TDABC estimate (orange diamond).

The intervals, however, carried information that a point estimate cannot. The support estimated from the first three years contained the recorded room occupancy time of 97.5% of the cases of the following two years (surgical time 97.5%, anesthesia time 97.4%, realized cost 97.8%); the α = 0.5 cut contained 76% and the α = 0.9 cut 24% (Figure 3a). Of the cases, 10.3% exceeded the α = 0.5 upper bound and 13.5% fell below its lower bound; α = 0.5 coverage was 73%, 87%, and 69% in Ranks 1–3. Figure 3b translates the intervals into a scheduling decision. Planning room time at the rule mean (the mean-based TDABC estimate) produced overruns in 43.6% of hold-out cases (mean overrun 34 min, mean idle time 28 min); the FL-TDABC centroid reduced overruns to 25.8% (19 min) at the cost of 62 min of idle time per case; the α = 0.5 upper bound to 10.3% (6 min) with 129 min idle; and the observed maximum almost eliminated overruns (1.1%) but left 285 min idle per case. The α scale thus traces a monotone trade-off between overrun risk and capacity utilization from which managers can choose.

### 5.5. Temporal Variation over the Study Period

Table 8 compares the five fiscal years. Case volume was lowest and the emergency share highest (23.6% versus 15.5–20.9% later) in FY2020, the first pandemic year; the share of Rank 3 procedures rose to 33.3% and 31.8% in FY2022–FY2023 and fell to 21.2% in FY2024, whereas the share of grades D and E was stable (70.9–74.5%). Median room occupancy time drifted from 204 to 184 min and differed across years (Kruskal–Wallis *p* = 0.038), surgical time did not (*p* = 0.21), and the dispersion of room time increased (coefficient of variation 0.58 to 0.65). Unit rates rose with salary revisions (surgeon ¥135 to ¥141, anesthesiologist ¥97 to ¥120 per minute), raising the mean realized cost from ¥337,984 to ¥387,521. The mean difference between FL-TDABC and realized cost ranged from 3.9% (FY2024) to 13.9% (FY2022) (*p* < 0.001), and recorded room time exceeded its rule median most in FY2020 (+28 min) and FY2024 (+34 min). Re-estimating the rules within each year reduced the difference to 1.8–9.8%, because the observed maximum of a rule grows with the number of cases from which it is taken. The department was renamed in April 2023 without any change in staff or services, and no discontinuity is visible at that point.

### 5.6. Surgical Complexity and Cost Uncertainty

**Figure 4:**
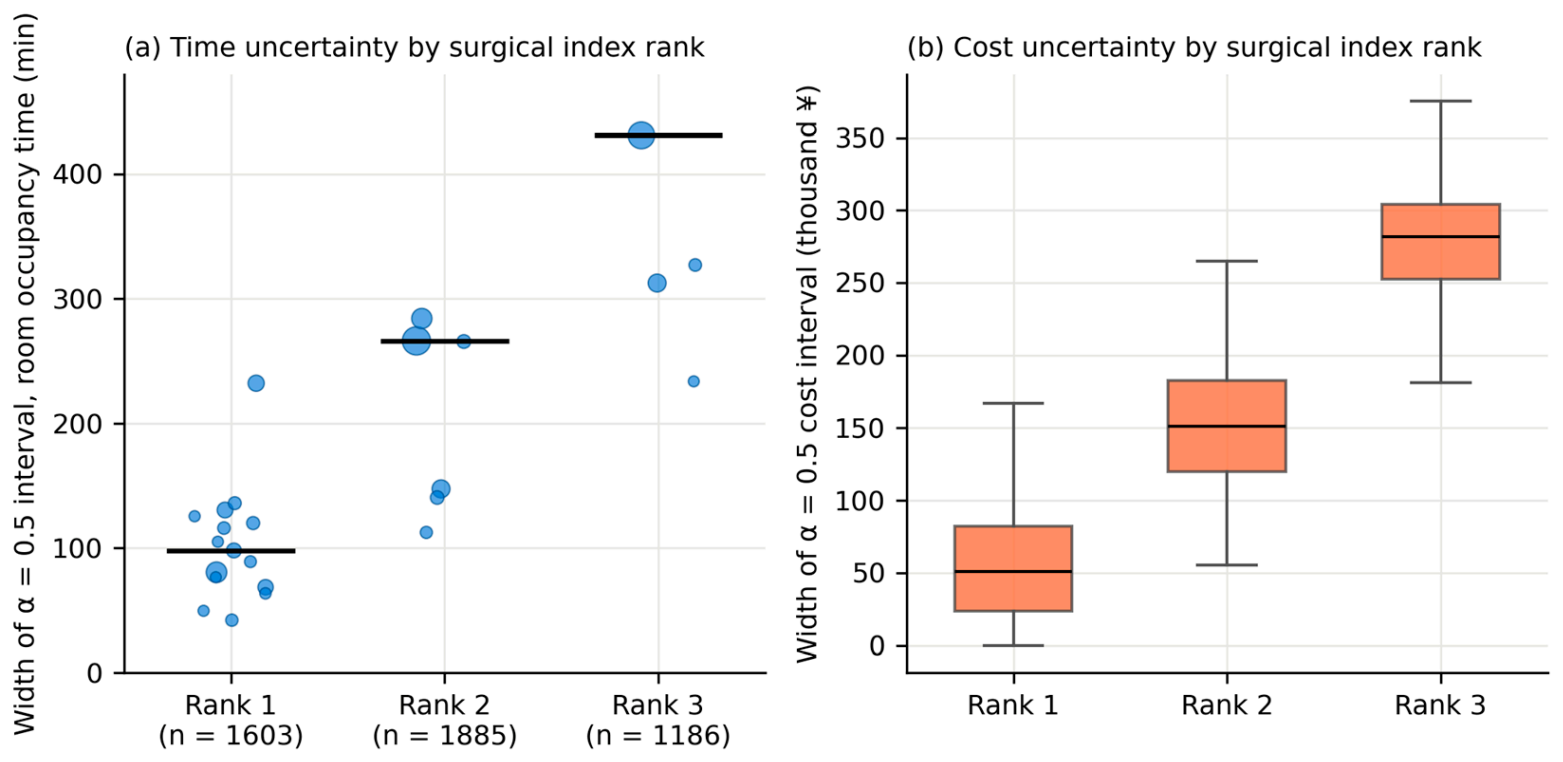
Cost uncertainty by surgical complexity. (**a**) Width of the α = 0.5 interval for room occupancy time by surgical index rank; dots are the 25 rules with at least 10 cases (marker size proportional to the number of cases) and bars the case-weighted median. (**b**) Width of the α = 0.5 cost interval per case by rank (boxes, interquartile range; whiskers, 1.5 × IQR; outliers omitted).

Figure 4 and Table 9 quantify the relationship between complexity and uncertainty. The median width of the α = 0.5 interval for room occupancy time rose from 98 min in Rank 1 to 266 min in Rank 2 and 431 min in Rank 3 (Kruskal–Wallis *p* < 0.001; Kendall’s τ = 0.81 at the case level; Spearman’s ρ = 0.79, *p* < 0.001, across the 25 rules with at least 10 cases), and the median width of the α = 0.5 cost interval increased from ¥50,781 to ¥151,052 and ¥281,748. The relationship held in absolute terms only: relative to estimated cost, interval width was similar across ranks (0.34, 0.43, and 0.38), and the coefficient of variation of recorded room time was highest in Rank 1 (0.52 versus 0.35 and 0.34). Complexity therefore raises the amount of money at risk, not the relative unpredictability of a case. The mean lower bound of the contribution margin (revenue minus the upper bound of the α = 0.5 cost interval) was positive in every rank, but at the case level it was negative for 39.5% of Rank 1, 24.8% of Rank 2, and 4.0% of Rank 3 cases (Table 9): financial risk from time variability is concentrated in simple, low-fee procedures, whose revenue leaves little room for a long case, whereas complex procedures carry the largest absolute uncertainty but also the largest margins.

### 5.7. Drivers of Contribution-Margin Variation

**Figure 5:**
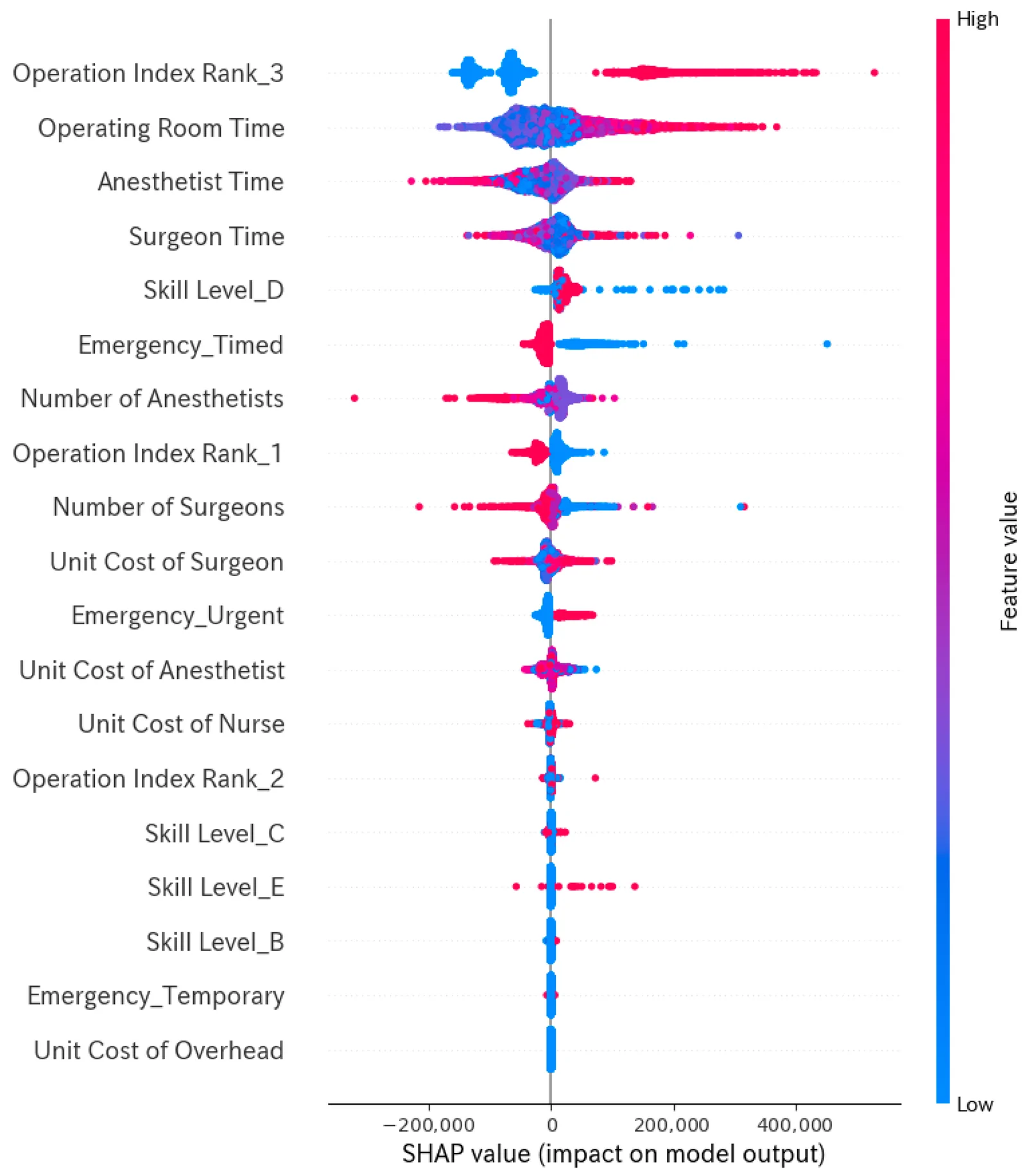
Shapley Additive exPlanation (SHAP) summary plot (variable impact on contribution margin). Note: Y-axis = variables; X-axis = SHAP value (impact). Red = high value; blue = low value.

SHAP analysis using a nonlinear XGBoost model identified key variables influencing case-level contribution margin (Figure 5). The most influential factor was the surgical index rank 3, highlighting the strong association between high-complexity surgeries and cost/profit fluctuations. Longer room occupancy and anesthesia times also adversely affected profitability, as did emergency procedures, which impose additional preparation and response costs. Time-related variables (room occupancy, anesthesia time, and surgical time) had a broader distribution of SHAP values than the unit-cost variables (surgeon and anesthesiologist rates). This partly reflects the structure of the data: unit rates are set annually by the hospital’s salary structure and vary only across fiscal years, whereas process times vary across every case, so the small contribution of unit costs is a feature of the setting rather than evidence that prices are unimportant.

Within a single institution, where unit costs are fixed by the salary structure and the national fee schedule, process time is the variable that operating room management can influence; the SHAP results therefore identify time as the lever available to managers—through scheduling, staffing, and process standardization—rather than establishing that time matters more than price in general.

At the level of individual rules, the α = 0.5 intervals for room occupancy time ranged from about 40 min to more than 400 min among the rules with at least 10 cases (Figure 4a). The widest belonged to scheduled grade D procedures of Rank 3 (431 min with one anesthesiologist, *n* = 796; 313 min with two, *n* = 288) and of Rank 2 (266–284 min), the narrowest to grade B and C procedures of Rank 1 without an anesthesiologist (42–81 min); rules with a single case have zero width by construction and carry no information about variability. From an operating room management perspective, the narrow-interval categories can be scheduled with minimal buffers, whereas the wide-interval categories indicate where explicit buffer time, flexible staffing, or pre-operative case review is needed.

Collectively, these findings indicate that FL-TDABC does not raise or lower costs across the board but attaches to each case a range whose position and width follow its rule; 19.3% of cases cost less under FL-TDABC than under TDABC, and the average premium can be positive, negligible, or negative depending on the specification (Section 5.3). What the model adds is the interval, whose empirical coverage (Section 5.4) and widening with complexity (Section 5.6) make it usable for cautious budgeting, scheduling buffers, and the identification of case groups with unplanned resource consumption.

Several practical challenges remain. Only 39 of the 96 possible rules occurred, and 14 contained fewer than 10 cases; pooling changed the results negligibly, but about 10 cases per rule is a reasonable minimum before a rule’s bounds are used for planning, and rules should be refreshed as cases accrue. The defuzzification method and the definition of the support—observed extremes or percentiles—should be chosen explicitly for the decision at hand, because they determine the size of the upper-tail allowance. Automated rule updating and integration with decision-support systems are natural extensions; their data-governance requirements are discussed in Section 6.

## 6. Discussion

This study expressed time-related variability in surgical cost estimation as interpretable intervals and examined both the point estimate and the intervals against realized costs. Three results define what the model does and does not offer. First, the higher average cost estimated by FL-TDABC (¥36,080 per case, 10.2%) is not an improvement in accuracy: out of sample, the fuzzy point estimate was biased upward and less accurate than a mean-based estimate, and the premium arises entirely from the maximum-time vertex. Second, the size and even the sign of the premium depend on the specification—observed extremes versus percentile bounds, and the defuzzification method—whereas the ordering of cases by cost does not; the aggregate ¥169 million over five fiscal years is the upper-tail allowance implied by planning on the full observed range, not a loss concealed by conventional costing. Third, the intervals have a defined empirical meaning: the observed range of each rule covered the recorded room occupancy time of 97.5% of the cases of the following two years and the α = 0.5 cut 76%, and the intervals widen systematically with complexity. FL-TDABC therefore complements point-based estimates by attaching a range with known coverage, and it is this range—not a higher point estimate—that constitutes decision-support information.

The theoretical contributions are fourfold. First, the effect of fuzzy uncertainty on a cost model is heterogeneous across case characteristics, as the effect sizes by grade and rank and the interval widths by rank and rule show. Second, the study explains a result reported but not explained in the FL-TDABC literature—that fuzzy costing yields higher or “more realistic” costs [12,17]: it is a mathematical consequence of anchoring the fuzzy number on the observed maximum and weighting it in the centroid, and it disappears under percentile supports, so higher fuzzy costs should not be read as hidden cost. Third, the model produces point estimates and intervals simultaneously, and it is the intervals that carry information surviving out-of-sample validation. Fourth, by treating the specification choices—representative value, support, membership function, defuzzification, and sparse-rule pooling—as objects of sensitivity analysis, the study provides a template for reporting FL-TDABC results.

From a managerial perspective, the intervals can be used in at least four ways. (1) Operating room scheduling. For scheduled grade D procedures of Rank 3 with two anesthesiologists (*n* = 288), recorded room occupancy time averaged 390 min, and planning at this mean would have overrun in about 40% of cases; planning at the α = 0.5 upper bound of the rule (568 min) reduced overruns to about 10%. Across all hold-out cases, the same planning policy cut overruns from 43.6% to 10.3% at the price of 129 min of idle time per case (Figure 3b); the α level can be set separately for high-volume elective lists, where idle time is costly, and for lists preceding emergency cover, where an overrun is costly. (2) Anesthesia staffing. For the same rule, the α = 0.5 upper bound of anesthesia time was 546 min against a mean of 368 min; the difference indicates how long a second anesthesiologist or overtime coverage must be available. (3) Surgical portfolio and case-mix management. Margin risk from time variability is concentrated in simple, low-fee procedures: for scheduled grade C procedures of Rank 1 without an anesthesiologist (*n* = 423), mean revenue was ¥97,222 against a realized cost of ¥84,270 and an α = 0.5 cost interval of ¥75,562–101,337, so the lower bound of the margin was negative (−¥4115); 45.7% of grade C Rank 1 cases and 50.6% of grade B cases had a negative lower-bound margin, against 4.0% of grade D Rank 3 cases. A department expanding low-fee day surgery should therefore monitor time variability more closely than one concentrating on complex procedures, whose margins absorb their larger absolute uncertainty. (4) Reimbursement and budgeting. Japanese hospitals cannot negotiate surgical fees, which are set nationally, but the rule-level time distributions correspond to the evidence that the surgical societies compile in the Gaihoren Shian to propose fee revisions [29,30,31], and within the hospital the intervals support risk-adjusted departmental budgets and the block-time allocation on which DPC/PDPS margins depend. In all four uses the interval—not the centroid—is the operative quantity, and the mean-based TDABC estimate remains the appropriate unbiased point estimate for accounting.

These uses map onto three dimensions of healthcare delivery performance. Efficiency: the α-cut intervals of the 39 observed case categories provide an evidence-based foundation for block times and buffer margins in operating room scheduling [7], and because unit costs are fixed within the institution, process time is the lever available to operating room management for improving contribution margins. Resilience: the intervals show where absolute cost uncertainty is largest (complex procedures) and where a long case turns the margin negative (simple, low-fee procedures), so that buffers and staffing can be planned where they matter, including the capacity reserved for emergency admissions, rather than overruns being absorbed after the fact. Value: linking cost variability to clinically interpretable drivers gives surgeons, anesthesiologists, and administrators a common language for discussing the cost of care, a prerequisite for the value-based healthcare that reimbursement reforms increasingly demand.

These implications extend previous FL-TDABC studies, which have been largely conceptual or applied outside the operating room [11,12], by demonstrating the approach at scale in surgical care, where both the magnitude of variability and its financial stakes are greatest.

Implementation requirements: data architecture and governance. The rule base can be refreshed automatically from the surgical management system, and machine learning could simplify rules or predict case duration in real time [34]. Such extensions would move the model from a retrospective analysis of anonymized extracts to a system processing identifiable clinical data continuously, and the corresponding requirements must be part of the design. In Japan, such systems must comply with the Ministry of Health, Labour and Welfare’s guidelines for the safety management of medical information systems [35] and the Act on the Protection of Personal Information, which require role-based access control, audit logging, encryption in transit and at rest, and de-identification or pseudonymization before data leave the clinical environment. An integrated platform for the surgical management system, the EHR, and the accounting system—for example, a lakehouse architecture, which unifies transactional records and analytical workloads under one governance layer [36]—would allow rules to be recalculated on schedule without duplicating identifiable data, and multi-institutional validation could rely on federated learning, which trains models across hospitals without pooling patient-level data [37]. The model needs only four categorical attributes and three time stamps per case, which limits the sensitive data involved; nevertheless, no real-time deployment should proceed without a privacy impact assessment and clinical governance approval.

Several limitations should be acknowledged. First, the analysis was conducted at a single acute-care hospital. Its anesthesia staffing model (14.7% of cases without an anesthesiologist, 6.0% with three), eight operating rooms with 24 h emergency cover, and the case mix of a designated cancer center determine which rules occur and their time distributions; other hospitals would need to re-estimate the TFNs from their own records, although the rule structure and validation procedure are transferable. Unit costs were derived from the hospital’s salary structure and a practical-capacity assumption of contracted working days × 7.5 h, without the allowance for non-productive time often recommended in TDABC, and operating room overhead, equipment depreciation, and administrative costs were not allocated, so absolute cost levels are understated and the comparison concerns direct costs only. Reimbursement and fee-schedule classification are national, but the hospital’s DPC coefficients and material procurement prices are not. Second, the fuzzy specification involves choices—representative value, support, membership function, and defuzzification—that determine the size of the point-estimate premium; results should be reported with a sensitivity analysis. Third, only 39 of the 96 possible rules occurred and 14 contained fewer than 10 cases; pooling did not change the results, but the coverage of sparse rules is less reliable. Fourth, the study period included the COVID-19 pandemic, salary revisions, and a renaming of the department; no discontinuity in case mix or process times was found, but the difference between fuzzy and realized costs varied by year, and rules estimated from five years of data carry a larger upper vertex than rules estimated from one year. Fifth, the validation benchmark was the realized time-driven cost computed from recorded process times, because case-level figures from the hospital’s departmental cost accounting were not available; the benchmark, like the fuzzy estimate, was computed by the authors from the recorded times, staffing, material costs, and the rates of Table 2, so the validation tests the process-time component only. Sixth, the α-cut intervals are possibility rather than probability intervals, and their empirical coverage should be re-estimated whenever the rules are refreshed. Finally, the SHAP analysis explains the model’s margin predictions within one institution and is not causal. Future research should extend FL-TDABC to multiple institutions and departments, examine how FL-TDABC-based buffers perform during disruptions, and compare the fuzzy intervals with those of probabilistic duration models.

## 7. Conclusions

This study developed an FL-TDABC model from 4674 surgical cases and validated it against realized costs. The higher average cost estimated by FL-TDABC (10.2%) arises from the maximum-time vertex of the triangular fuzzy number, depends on the support and the defuzzification method, and does not represent improved accuracy; out of sample, the fuzzy point estimate was less accurate than a mean-based estimate. The intervals, in contrast, had stable empirical properties: the observed range of a rule covered the recorded room occupancy time of 97.5% of cases in the following two years and the α = 0.5 cut 76%, interval width increased with complexity, and the risk of a negative margin concentrated in simple, low-fee procedures. Planning room time at the α = 0.5 upper bound would have reduced overruns from 44% to 10% of cases at the cost of idle time, so the α level can serve as an explicit setting for the trade-off between overrun risk and capacity utilization. FL-TDABC therefore complements, rather than replaces, conventional TDABC by adding a range with known coverage to a point estimate; whether acting on this range improves resource allocation is a question for prospective evaluation that this retrospective study cannot answer. Because the rules use four routinely recorded attributes, the approach adds no measurement burden, provided that its specification is reported with a sensitivity analysis and that any automated deployment meets the data-governance requirements discussed above. Multi-institutional validation and comparison with probabilistic duration models remain for future research.

## Figures and Tables

**Table 1:** Rule variables: definition, levels, and clinical and economic relevance.

Variable	Definition and Source	Levels	Clinical Relevance	Economic Relevance
Surgical index rank	Composite index of typical duration, personnel, and complexity from the Gaihoren Shian [29,30,31], assigned by procedure code	Rank 1 (<14.10); Rank 2 (14.10–31.73); Rank 3 (≥31.73)	Expected magnitude of the operation and of its resource demand	Correlates with the fixed surgical fee and with expected labor and room time
Technical grade	Gaihoren classification of the skill level required for the procedure	B, C, D, E	Procedural specialization and risk	Basis of fee-schedule proposals; higher grades carry higher fees and material costs
Number of anesthesiologists	Anesthesiologists assigned to the case, from the surgical management system	0, 1, 2, 3	Anesthesia staffing model (0 = local or regional anesthesia by the surgical team)	Directly determines anesthesia labor cost per minute
Urgency	Scheduling status recorded at registration	Scheduled; Other (emergency, ad hoc)	Unplanned cases with preparation and off-hours burden	Off-hours surcharges in revenue; overtime and disruption costs

**Table 2:** Capacity cost rates by fiscal year (¥ per minute) and practical-capacity assumptions.

Fiscal Year	Surgeon Rate	Anesthesiologist Rate	Nurse Rate	Working Days	Practical Capacity per FTE (min/year)
FY2020	135	97	51	244	109,800
FY2021	133	110	51	243	109,350
FY2022	135	112	52	244	109,800
FY2023	140	113	51	244	109,800
FY2024	141	120	52	243	109,350

Note: Rate = annual personnel expense of the pool ÷ (full-time-equivalent staff × working days × 7.5 h × 60 min), rounded to the nearest yen. Surgeon rates for FY2020–FY2022 are the weighted average of the general surgery group (gastrointestinal, breast, and thoracic surgery) under which the department was administered; FY2023–FY2024 rates are those of the Gastrointestinal Surgery department. Nurse rates refer to operating room nurses. Overhead was not allocated.

**Table 3:** Distribution of the 4674 cases across rule variables, setting, and fiscal year.

Variable	Category	*n*	%
Surgical index rank	Rank 1 (<14.10)	1603	34.3
	Rank 2 (14.10–31.73)	1885	40.3
	Rank 3 (≥31.73)	1186	25.4
Technical grade	B	259	5.5
	C	1025	21.9
	D	3372	72.1
	E	18	0.4
Anesthesiologists	0	686	14.7
	1	2647	56.6
	2	1059	22.7
	3	282	6.0
Urgency	Scheduled	3774	80.7
	Emergency	893	19.1
	Ad hoc	7	0.1
Setting	Inpatient	3912	83.7
	Outpatient	762	16.3
Fiscal year	FY2020	890	19.0
	FY2021	980	21.0
	FY2022	937	20.0
	FY2023	952	20.4
	FY2024	915	19.6

**Table 4:** Comparison of cost estimates between the time-driven activity-based costing (TDABC) and fuzzy-logic time-driven activity-based costing (FL-TDABC) models.

Technical Grade	*n*	TDABC Mean Cost (¥)	FL-TDABC Mean Cost (¥)	Difference (¥)	Difference (%)	Cohen’s d
B	259	32,415.73	38,655.38	6239.65	19.25	0.83
C	1025	165,184.96	182,144.35	16,959.39	10.27	0.53
D	3372	434,481.49	478,801.41	44,319.92	10.20	0.61
E	18	880,464.72	891,080.06	10,615.33	1.21	0.13
Overall	4674	354,863.13	390,942.97	36,079.84	10.17	0.558

**Table 5:** Comparison of cost estimates and contribution margins between the TDABC and FL-TDABC models by technical grade and surgical index rank.

Grade	Rank	*n*	TDABC Mean Cost (¥)	FL-TDABC Mean Cost (¥)	Difference (¥)	Cohen’s d	Margin, TDABC (¥)	Margin, FL-TDABC (¥)
B	Rank 1	259	32,415.73	38,655.38	6239.65	0.83	17,494.92	11,255.27
C	Rank 1	1020	162,969.48	180,238.83	17,269.35	0.56	54,609.50	37,340.15
C	Rank 2	5	617,142.80	570,870.40	−46,272.40	−0.47	−9100.80	37,171.60
D	Rank 1	324	224,581.94	234,259.86	9677.93	0.34	126,109.79	116,431.86
D	Rank 2	1880	314,294.75	353,655.07	39,360.31	0.78	192,731.10	153,370.79
D	Rank 3	1168	686,158.31	748,070.76	61,912.45	0.62	459,035.10	397,122.65
E	Rank 3	18	880,464.72	891,080.06	10,615.33	0.13	939,360.83	928,745.50

Note: Margin = contribution margin (mean surgical revenue − mean estimated cost); the mean revenue of each group is identical under both models. Difference = FL-TDABC − TDABC. Cohen’s d is computed on the paired case-level differences. Grade = technical grade; Rank = surgical index rank.

**Table 6:** Sensitivity of the FL-TDABC results to the specification of the fuzzy numbers (*n* = 4674).

Specification	Mean FL-TDABC Cost (¥)	Mean Difference (¥)	Difference (%)	Cases with FL-TDABC > TDABC (%)	Cohen’s d	ρ, Costs vs. Base	ρ, Differences vs. Base
Base: mode; observed min–max; centroid	390,943	36,080	10.2	80.7	0.558	1.000	1.000
Representative value: median	396,645	41,782	11.8	82.8	0.634	1.000	0.995
Representative value: 10% trimmed mean	398,134	43,271	12.2	83.2	0.656	1.000	0.994
Representative value: mean	399,750	44,887	12.6	83.7	0.677	1.000	0.992
Support: 5th–95th percentiles	354,165	−699	−0.2	58.7	−0.012	0.994	0.840
Support: 10th–90th percentiles	348,272	−6591	−1.9	53.4	−0.109	0.993	0.801
Support: 2.5th–97.5th percentiles	360,207	5344	1.5	64.4	0.089	0.995	0.876
Membership: trapezoidal (plateau 25th–75th percentiles)	394,269	39,406	11.1	81.9	0.606	1.000	0.997
Defuzzification: weighted average (*a* + 2*m* + *b*)/4	374,853	19,990	5.6	73.7	0.323	0.999	0.977
Defuzzification: bisector of area	381,070	26,207	7.4	76.6	0.417	1.000	0.992
Defuzzification: mean of maximum (=mode)	326,583	−28,280	−8.0	33.4	−0.434	0.987	0.584
Perturbation: upper vertex × 0.8	367,739	12,876	3.6	68.3	0.207	1.000	0.963
Perturbation: upper vertex × 1.2	414,147	59,283	16.7	88.6	0.846	1.000	0.976
Perturbation: mode × 0.9	387,602	32,739	9.2	79.1	0.509	1.000	0.999
Perturbation: mode × 1.1	394,284	39,421	11.1	82.4	0.606	1.000	0.999
Perturbation: lower vertex × 0.8	387,910	33,047	9.3	79.4	0.513	1.000	0.999
Perturbation: lower vertex × 1.2	393,976	39,112	11.0	82.2	0.602	1.000	0.999
Perturbation: all vertices × 0.9	374,484	19,621	5.5	72.7	0.312	1.000	0.981
Perturbation: all vertices × 1.1	407,402	52,539	14.8	86.8	0.773	1.000	0.986
Pooling: rules with *n* < 10 pooled with parent group	391,172	36,309	10.2	80.8	0.558	1.000	0.992
Pooling: rules with *n* < 30 pooled with parent group	390,897	36,034	10.2	80.7	0.549	1.000	0.988
Pooling (*n* < 10) + 5th–95th percentile support	354,142	−721	−0.2	58.9	−0.012	0.994	0.834

Note: Difference = FL-TDABC − realized TDABC cost; ρ = Spearman rank correlation between case-level values under the alternative and the base specification. Mean realized TDABC cost = ¥354,863 in all rows.

**Table 7:** Out-of-sample validation against realized TDABC costs: temporal hold-out (rules from FY2020–FY2022 applied to FY2023–FY2024, *n* = 1867) and 5-fold cross-validation (mean of folds).

Metric	Temporal Hold-Out	5-Fold Cross-Validation
Cases (training/test)	2807/1867	3739/935 per fold
Mean-based TDABC estimate: bias (¥)	−7944	−2010
Mean-based TDABC estimate: MAE (¥)	42,515	38,426
Mean-based TDABC estimate: RMSE (¥)	68,810	61,141
Mean-based TDABC estimate: median absolute percentage error (%)	9.2	8.9
Median-based estimate: bias (¥)	−16,707	−11,382
Median-based estimate: MAE (¥)	42,551	37,880
Median-based estimate: RMSE (¥)	71,555	63,040
Median-based estimate: median absolute percentage error (%)	8.7	8.6
FL-TDABC centroid: bias (¥)	23,024	34,136
FL-TDABC centroid: MAE (¥)	52,938	53,546
FL-TDABC centroid: RMSE (¥)	73,134	73,163
FL-TDABC centroid: median absolute percentage error (%)	13.4	14.2
Coverage of room occupancy time: support/α = 0.5/α = 0.9 (%)	97.5/76.2/23.5	98.7/82.9/26.0
Coverage of realized cost: support/α = 0.5/α = 0.9 (%)	97.8/76.5/23.5	98.7/84.6/27.8
Room time planned at rule mean: overrun rate (%)/mean overrun (min)/mean idle (min)	43.6/34/28	42.3/29/29
Room time planned at FL-TDABC centroid: overrun rate (%)/mean overrun (min)/mean idle (min)	25.8/19/62	20.4/13/72
Room time planned at α = 0.5 upper bound: overrun rate (%)/mean overrun (min)/mean idle (min)	10.3/6/129	5.7/3/153
Room time planned at α = 0.9 upper bound: overrun rate (%)/mean overrun (min)/mean idle (min)	42.6/36/30	40.9/28/34
Room time planned at observed maximum: overrun rate (%)/mean overrun (min)/mean idle (min)	1.1/0/285	0.7/0/330

Note: bias = mean of (estimate − realized cost); MAE = mean absolute error; RMSE = root mean squared error; support = observed minimum–maximum of the rule. Rules with fewer than 10 training cases were pooled with their parent group; two rules (five cases) unobserved in the training period received pooled parameters.

**Table 8:** Case mix, process times, unit rates, and cost estimates by fiscal year.

	FY2020	FY2021	FY2022	FY2023	FY2024
Cases (*n*)	890	980	937	952	915
Emergency or ad hoc cases (%)	23.6	20.9	15.5	18.0	18.5
Rank 3 cases (%)	20.1	20.2	33.3	31.8	21.2
Grade D or E cases (%)	71.9	74.5	72.6	70.9	72.7
Median surgical time (min)	133	127	130	120	124
Median anesthesia time (min)	189	177	182	174.5	175
Median room occupancy time (min)	203.5	188.5	191	186	184
CV of room occupancy time	0.58	0.55	0.60	0.63	0.65
Surgeon/anesthesiologist/nurse rate (¥/min)	135/97/51	133/110/51	135/112/52	140/113/51	141/120/52
Mean realized TDABC cost (¥)	337,984	336,692	357,756	355,113	387,521
Mean FL-TDABC cost (¥)	364,655	375,525	407,532	403,979	402,475
Mean difference (¥)	26,670	38,833	49,776	48,866	14,954
Difference (%)	7.9	11.5	13.9	13.8	3.9
Cases with FL-TDABC > TDABC (%)	78.2	84.8	85.5	85.4	69.1
Difference with rules re-estimated within the year (%)	6.0	4.8	6.4	9.8	1.8

Note: Fiscal years run from April to March. CV = coefficient of variation. Rates are the capacity cost rates of Table 2. Difference = FL-TDABC − realized TDABC cost; rules are estimated from all five years except in the last row.

**Table 9:** Interval width, dispersion of recorded times, and margin risk by surgical index rank.

	Rank 1	Rank 2	Rank 3
Cases (*n*)	1603	1885	1186
Median width of α = 0.5 interval, room occupancy time (min)	98	266	431
Mean width of observed range (support), room occupancy time (min)	222	481	777
Median width of α = 0.5 cost interval (¥)	50,781	151,052	281,748
Median relative width of α = 0.5 cost interval (width ÷ FL-TDABC cost)	0.34	0.43	0.38
CV of recorded room occupancy time	0.52	0.35	0.34
SD of recorded room occupancy time (min)	61	73	129
Mean difference FL-TDABC − TDABC (¥)	13,953	39,133	61,134
Mean lower/upper bound of the contribution margin under the α = 0.5 cost interval (¥)	25,717/83,495	95,394/246,237	293,780/572,139
Cases with a negative lower bound of the contribution margin (%)	39.5	24.8	4.0

Note: Interval widths are constant within a rule and are summarized across cases. The lower (upper) bound of the contribution margin is revenue minus the upper (lower) bound of the α = 0.5 cost interval.

## Data Availability

The datasets presented in this article are not readily available because they contain confidential hospital- and patient-level information provided by the participating hospital. Requests to access the datasets should be directed to the corresponding author.

## Abbreviations

TDABC	Time-Driven Activity-Based Costing
FL-TDABC	Fuzzy-Logic Time-Driven Activity-Based Costing
VBHC	Value-Based Healthcare
ABC	Activity-Based Costing
DPC	Diagnosis Procedure Combination
PDPS	Per Diem Payment System
TFN	Triangular Fuzzy Numbers
SHAP	Shapley Additive exPlanation
FY	Fiscal Year (April–March)
MAE	Mean Absolute Error
RMSE	Root Mean Squared Error
EHR	Electronic Health Record
CV	Coefficient of Variation
IQR	Interquartile Range
PFABC	Performance-Focused Activity-Based Costing
AI	Artificial Intelligence

## Appendix A

**Table A1:** Triangular fuzzy numbers (*a*, *m*, *b*) of the 39 observed rules: minimum, mode, and maximum of the recorded surgical, anesthesia, and room occupancy times (minutes) and number of cases.

Rule	*n*	Surgical Time (*a*, *m*, *b*)	Anesthesia Time (*a*, *m*, *b*)	Room Occupancy Time (*a*, *m*, *b*)
1_B_0_Scheduled	170	4, 9, 113	5, 39, 114	15, 50, 152
1_B_0_Other	58	6, 23, 67	9, 10, 77	19, 37, 103
1_B_1_Scheduled	18	25, 35, 72	70, 77, 127	75, 90, 174
1_B_1_Other	4	32, 46, 48	56, 97, 98	65, 108, 110
1_B_2_Scheduled	8	42, 46, 108	79, 113, 159	85, 119, 167
1_B_3_Scheduled	1	38, 38, 38	79, 79, 79	87, 87, 87
1_C_0_Scheduled	423	4, 32, 163	12, 52, 165	28, 69, 189
1_C_0_Other	28	14, 25, 138	26, 69, 139	43, 77, 170
1_C_1_Scheduled	206	23, 65, 443	57, 130, 498	68, 158, 532
1_C_1_Other	189	20, 63, 247	50, 121, 307	59, 131, 320
1_C_2_Scheduled	78	16, 38, 249	56, 141, 318	63, 136, 335
1_C_2_Other	66	13, 45, 220	73, 102, 313	92, 134, 324
1_C_3_Scheduled	9	36, 36, 263	109, 109, 318	118, 118, 326
1_C_3_Other	21	43, 43, 254	93, 99, 310	106, 110, 357
1_D_0_Scheduled	4	31, 42, 86	48, 62, 133	72, 83, 140
1_D_0_Other	2	19, 19, 54	25, 25, 54	30, 30, 89
1_D_1_Scheduled	151	38, 96, 211	73, 114, 258	87, 154, 283
1_D_1_Other	44	30, 105, 201	77, 138, 253	83, 153, 261
1_D_2_Scheduled	84	30, 149, 265	73, 179, 321	89, 133, 329
1_D_2_Other	20	21, 109, 218	62, 62, 269	69, 69, 279
1_D_3_Scheduled	7	53, 182, 237	96, 252, 297	107, 255, 310
1_D_3_Other	12	54, 97, 205	106, 161, 260	113, 169, 266
2_C_1_Scheduled	4	159, 159, 430	223, 223, 546	243, 243, 566
2_C_3_Scheduled	1	214, 214, 214	325, 325, 325	337, 337, 337
2_D_0_Scheduled	1	102, 102, 102	174, 174, 174	177, 177, 177
2_D_1_Scheduled	925	33, 95, 513	75, 150, 587	84, 152, 616
2_D_1_Other	287	19, 102, 298	77, 203, 368	94, 154, 389
2_D_2_Scheduled	405	31, 82, 574	81, 161, 644	98, 139, 666
2_D_2_Other	100	54, 112, 303	98, 162, 372	102, 177, 383
2_D_3_Scheduled	109	55, 92, 530	92, 155, 617	110, 251, 641
2_D_3_Other	53	64, 142, 270	95, 195, 334	112, 196, 337
3_D_1_Scheduled	796	88, 207, 907	126, 299, 995	148, 337, 1010
3_D_1_Other	7	138, 195, 243	180, 243, 291	193, 308, 308
3_D_2_Scheduled	288	86, 177, 660	132, 324, 768	156, 354, 781
3_D_2_Other	8	117, 163, 415	199, 215, 486	212, 223, 491
3_D_3_Scheduled	68	127, 388, 746	175, 300, 821	191, 356, 845
3_D_3_Other	1	243, 243, 243	329, 329, 329	330, 330, 330
3_E_1_Scheduled	16	199, 499, 637	270, 613, 728	287, 633, 754
3_E_2_Scheduled	2	555, 572, 572	644, 664, 664	660, 689, 689

Note: Rule = surgical index rank_technical grade_number of anesthesiologists_urgency (Scheduled or Other). For rules with a single case the three vertices coincide. Where no value recurs within a rule, the mode is the first observed value (Section 4.3); the modes of the three times are computed independently and need not be ordered. Base specification (observed minimum, mode, and maximum); rules with fewer than 10 cases were pooled with their parent group in the sensitivity and validation analyses (Section 4.3).
